# What influences women’s movement and the use of different positions during labour and birth: a systematic review protocol

**DOI:** 10.1186/s13643-018-0857-8

**Published:** 2018-11-13

**Authors:** Helen L. Watson, Alison Cooke

**Affiliations:** 1 Sheffield Teaching Hospitals NHS Foundation Trust, Jessop Wing, Tree Root Walk, Sheffield, S10 2SF UK; 2 Division of Nursing, Midwifery and Social Work, School of Health Sciences, Faculty of Biology, Medicine and Health, The University of Manchester, Jean McFarlane Building Room 4.336, Oxford Road, Manchester, M13 9PL UK

**Keywords:** Systematic review, Protocol, Movement, Position, Labour, Birth

## Abstract

**Background:**

Women want to give birth in a safe and supportive environment where they are free to move and adopt different positions. Moving freely and using different positions in labour results in a range of physical and psychological benefits for women. However, many women report that they are restricted from moving freely during labour and birth and it is important to understand the factors that are influencing this.

**Methods:**

A mixed-methods systematic review will be undertaken. Qualitative, quantitative and mixed-methods primary empirical studies will be identified by systematically searching seven electronic databases using a search strategy that includes medical subject headings (MeSH) and keywords to cover synonyms and related terms. In addition, reference-tracking will be undertaken, and expert researchers will be contacted to locate relevant studies. Two reviewers will be involved in the assessment of the studies against eligibility criteria, formal quality appraisal and data extraction. A results-based convergent synthesis will be undertaken, using narrative synthesis if the quantitative data are too heterogeneous for meta-analysis, meta-ethnography for the synthesis of the qualitative data and the production of a line of argument synthesis. Finally, confidence in the findings will be formally assessed and conclusions drawn.

**Discussion:**

The findings of this review will allow researchers, practitioners and policy makers to better understand the factors influencing women’s movement and the use of different positions during labour and birth. This will inform future research and the development of maternity services designed to implement best-evidence concerning movement and positioning during labour and birth into clinical practice.

**Systematic review registration:**

In accordance with the PRISMA-P guidelines (Moher et al. Syst Rev 4:1, 2015), the systematic review protocol was registered with the International Prospective Register of Systematic reviews (PROSPERO) on July 17, 2018 (CRD42018103354).

**Electronic supplementary material:**

The online version of this article (10.1186/s13643-018-0857-8) contains supplementary material, which is available to authorized users.

## Background

The recently published World Health Organization (WHO) recommendations regarding intrapartum care for a positive childbirth experience [1] highlight that women want to give birth in a safe and supportive environment where they are free to move and adopt different positions. There is a large body of evidence which demonstrates that freedom to move and adopt upright positions in labour results in a range of physical and psychological benefits for women, including reduced risk of caesarean section, increased agency and sense of control during labour and increased satisfaction with the birth experience [2, 3].

Restricting women’s movement during labour and preventing women from adopting comfortable positions is described within a group of poor healthcare professional behaviours constituting disrespect, abuse and mistreatment during maternity care [4]. This is widely criticised as contravening childbearing women’s rights to be treated with dignity, to be free from harm and ill treatment and to have their choices and preferences respected [5].

The WHO therefore recommends that women should be encouraged to be mobile and to adopt comfortable positions of their choice, including upright positions, during labour and birth, but emphasises that particular positions should not be forced on women [1]. However, several recent national surveys have demonstrated that despite national and international guidance and professional governing body recommendations [1, 6–12], large numbers of women across the world continue to give birth lying down with or without their feet in stirrups; 50% in England and Wales [13], 68% in the United States of America (USA) [14], 47.9% in Canada [15] and 92% in Brazil [16], and significant numbers of women report they are not free to move or change position during some or all of their labour; 30% in England and Wales [13], 55% in Brazil [16] and 57% in the USA [14].

Understanding factors that influence women’s movement and the use of different positions during labour and birth is crucial for the provision of quality, women-centred and human-rights based maternity healthcare services. However, there remains a lack of systematic evidence synthesis concerning this issue.

### Objectives

The review aims to synthesise the evidence concerning women’s freedom of movement and the use of different positions during labour and birth. The review will address the following question:

- What factors influence women’s movement and the positions that they adopt during labour and birth?

## Methods

The protocol has been developed following the preferred reporting items for systematic review and meta-analysis protocols (PRISMA-P) statement [17]. The PRISMA-P checklist is included in Additional file 1. The review protocol has been registered with PROSPERO (CRD42018103354). The enhancing transparency in reporting the synthesis of qualitative research (ENTREQ) statement [18] has also informed the development of the protocol.

### Methodology

The systematic review will apply the principles of mixed-methods research to integrate results from qualitative, quantitative and mixed-methods studies [19]. The retrieval of qualitative and quantitative data within a review can maximise the usefulness of the synthesis by providing an understanding of human experience alongside empirical evidence about a particular phenomenon [20], in this case, factors influencing movement and the use of different positions during labour and birth.

### Eligibility criteria

Studies will be selected according to the following criteria.

#### Types of studies

Only peer-reviewed, published, qualitative, quantitative or mixed-methods studies will be included. Review articles will be excluded; however, they will be used to cross check for relevant primary empirical studies. If a review addressing a similar question is identified, the review team will consider relevance and quality when deciding whether to include all previously reviewed studies or to amend the protocol to undertake an update of the review. Conference abstracts will be excluded; however, where the content appears relevant searches for papers reporting the full study will be undertaken including a maximum of two attempts to contact the authors by email. Editorials will be excluded. Studies will be included regardless of length of time of follow-up or length of time since the experience occurred. Studies in any setting will be included. Only studies in English will be included; however, a list of possibly relevant studies in other languages will be provided as an appendix in the final review.

#### Types of participants

Women of any age experiencing labour and birth in any setting will be included. Women labouring with babies in the breech position will be excluded. Women who are restricted from moving during labour and birth due to pre-existing medical conditions or disabilities will be excluded, as will women who are restrained due to being detained by the state as prisoners or under criminal investigation and women experiencing obstetric emergencies. Studies including heath care providers or women’s family members or birth supporters will also be included if they provide relevant data pertaining to factors influencing women’s movement and positioning during labour and/or birth.

#### Types of interventions

Whilst this review is not focussing on evidence about interventions, studies investigating interventions will be included if they meet the eligibility criteria.

#### Types of comparators

Studies do not have to include a comparator to be eligible for inclusion.

#### Types of outcome measures

Factors influencing maternal movement and/or positions adopted during labour and birth from the perspective of the labouring woman, health professionals, family members or birth supporters will be included.

### Information sources

A search strategy will be conducted to identify studies meeting the inclusion criteria. The following electronic databases will be searched to identify relevant studies:

- The Cumulative Index to Nursing and Allied Health Literature (CINAHL) via EBSCO (1982 onwards)
- Medical Literature Analysis and Retrieval System Online (MEDLINE) via Ovid (1946 onwards)
- Maternal and Infant Care via Ovid (1971 onwards)
- PsycINFO via Ovid (1806 onwards)
- Applied Social Sciences Index & Abstracts (ASSIA) via ProQuest (1987 onwards)
- International Prospective Register of Systematic reviews (PROSPERO)
- African Journals Online (AJOL)

In addition to the electronic database search, the reference lists of eligible studies will be examined to identify any other relevant studies, and midwifery researchers will be contacted via the JISCMail midwifery research email group and asked to identify any further relevant studies.

### Search strategy

The development of the search strategy will be an iterative process undertaken by the review team and will include scoping exercises across several databases. The search terms will consist of three broad strings covering population, phenomenon of interest and context [21]. Medical subject headings (MeSH) and keywords will be used to cover synonyms and related terms, and wildcards and truncation functions will be used to ensure the search is as comprehensive as possible [22, 23]. Search terms will be combined using Boolean operators. The search strategy will be developed and agreed by the review team. The MEDLINE search strategy is included in Additional file 2. The search terms will be adapted to the syntax and subject headings for all other identified databases.

### Study records

#### Data management

Literature searching will be undertaken by HW, the number of records retrieved from the searches will be recorded, and the citations will be imported into the electronic bibliographic software Endnote X8.2 (Clarivate Analytics).

#### Selection process

Duplicate records will be removed in Endnote, and the remaining records will be imported into Microsoft Excel and screened against the pre-defined eligibility criteria. One reviewer (HW) will conduct the initial screening based on title and abstract, and the second reviewer will screen 10% of these records. Full-text versions of any records deemed potentially eligible for inclusion by either reviewer will then be screened by two reviewers (HW and 2nd reviewer). Disagreements between the reviewers during the initial or full screening stage will be resolved through discussion or deferral to a third reviewer (AC) if agreement cannot be reached. If there is less than 90% agreement between the two authors during initial screening, then both authors will independently assess all the retrieved records. Reasons for exclusion of studies at the full-text stage will be recorded and provided as an additional file in the final review. A PRISMA diagram [24] will be provided to show the study search and assessment process.

### Quality appraisal

As this review will include qualitative, quantitative and mixed-methods primary studies, the Mixed Methods Appraisal Tool (MMAT) [25] will be used to appraise the quality of the methods of each included study. This tool includes criteria for all study types and has established content validity [26, 27]. Two reviewers (HW and 2nd reviewer) will critically appraise the quality of the included studies; disagreements will be discussed and referred to the third reviewer (AC) if they cannot be resolved. A descriptive summary of the quality of each study will be presented in the final review report, along with an overall quality score calculated using the MMAT scoring metrics and presented as follows: (*) one criterion met to (****) all criteria met [25]. No studies will initially be excluded on the basis of the quality assessment as it is envisaged that only a small volume of relevant literature will be identified; however, the quality scores will be used to inform the confidence of the review findings (detail is provided in the ‘Data Synthesis’ section).

### Data items

Data extraction will be managed in Microsoft Excel using a data extraction proforma developed specifically for this review which will be piloted by two reviewers (HW and 2nd reviewer) on 2 studies (see Additional file 3). Data to be extracted will include publication information, study characteristics, participant information and outcomes [28]:

- Publication information: study title, authors, journal title, year of publication, sources of funding
- Study characteristics: aim of study and/or research questions, study type, theoretical perspective, setting, sample size, and methods of sampling, recruitment, data collection and analysis
- Participant information: age, number of children, ethnicity, inclusion/exclusion criteria
- Outcomes: Factors influencing freedom of movement or the use of different positions during labour or birth

In the case of missing information, one reviewer (HW) will attempt to contact the authors to retrieve this with a maximum of two email attempts. The extracted data will be summarised, tabulated and presented in the final review report. Outcome data will be extracted from sections of the quantitative studies entitled “results” and “findings”. An inclusive approach will be taken with the qualitative studies [29], whereby outcomes will be extracted from the “findings” or “results” sections of the papers as well as the author interpretations within the discussion/interpretation/conclusion sections of the studies. Qualitative data will be extracted directly into NVivo-11 software (QSR International). Data extraction will be undertaken by the first reviewer (HW), and a second reviewer will assess the accuracy of the extracted data against the original studies. Any disagreements will be discussed and, if not resolved, referred to the third reviewer (AC). In this review, factors influencing freedom of movement or positioning in labour will be considered to be the main outcomes and no other outcomes will be included.

### Data synthesis

As this review will comprise data from mixed study methods, a results-based convergent synthesis will be undertaken [30], based on the segregated method proposed by Sandelowski et al. [31]. This method is based on the assumptions that qualitative and quantitative studies can be distinguished from each other and that the differences between them justify separate analyses using synthesis methods designed specifically for the data type [31]. The separate synthesis methods are described below, and once completed, the synthesis products will then be synthesised themselves [31], by comparing and/or juxtaposing the findings from the qualitative and quantitative evidence [30] and forming a final line of argument synthesis [32].

#### Synthesis of quantitative data

If there is sufficient clinical heterogeneity to expect that the underlying treatment effects differ between trials, or if we detect substantial statistical heterogeneity using the *I*^2^ statistic, meta-analysis will not be appropriate, and a narrative synthesis of all quantitative study types will be undertaken. We will seek statistical advice for this part of the analysis if appropriate. Narrative synthesis will be based on guidance developed by Popay et al. [33] and as recommended by a Cochrane Review Group [34] and The Centre for Reviews and Dissemination (CRD) [22]. This will involve developing a preliminary synthesis, exploring relationships within and between the studies and assessing the robustness of the synthesis [33]. Assessment of the robustness of the synthesis will inform the assessment of confidence in the findings and will include the quality assessment scores of the individual studies, an assessment of the Grading of Recommendations Assessment, Development and Evaluation (GRADE) criteria [35] where these can be applied appropriately [36], and critical reflection on the synthesis process [22, 34].

#### Synthesis of qualitative data

As the intention of the qualitative synthesis is interpretation and to generate new theoretical insights, meta-ethnography, as first described by Noblit and Hare [32], has been chosen as the most appropriate method to facilitate this [28, 37]. This method is also appropriate when synthesising data from studies that have used different qualitative methodologies [37]. The synthesis will comprise the following steps based on meta-ethnography as proposed by Noblit and Hare [32]: reading the studies, identifying findings from one paper and comparing them to findings from another and generating a list of concepts. Next will follow the use of reciprocal translation, the identification of similarities in the concepts and developing themes and refutational translation which is the search for disconfirming or unexplained data, and finally, there will be synthesis of the themes into a “line of argument” synthesis, which is a summary statement of the findings and theoretical insights [32].

The confidence in the qualitative findings will be assessed using the Confidence in the Evidence from Reviews of Qualitative Research (CERQual) approach [38]. This includes four elements: methodological limitations of the individual studies, relevance to the review question, coherence and adequacy of the data. The overall CERQual assessment score ranging from high confidence to very low confidence of each finding will be made through discussion among all review authors [39]. The CERQual assessment of each synthesis finding will be provided as an appendix to the final review and will be summarised in a CERQual evidence summary table [40].

## Discussion

Several systematic reviews have applied meta-analysis to the data reported about outcomes associated with movement and positions adopted during labour and birth [2, 3]. This has led to the publication of multiple national and international guidelines encouraging mobility and the use of comfortable positions of a woman’s choice during labour and birth [1, 6–12]. National level data indicates ongoing restriction of women’s movement during labour and birth; however, there is a lack of evidence synthesis related to factors that contribute to this. This review will comprehensively and formally synthesise the available primary quantitative, qualitative and mixed-methods studies reporting data relating to this topic. The findings of this review will allow researchers, practitioners and policy makers to better understand the barriers and facilitators influencing women’s movement and the use of different positions during labour and birth. This will inform future research and the development of services designed to implement best-evidence concerning movement and positioning during labour and birth into clinical practice.

## Additional files

Additional file 1: PRISMA-P checklist. Completed PRISMA-P checklist for this review protocol. (DOCX 23 kb)

Additional file 2: MEDLINE search terms. Terms to be used when searching MEDLINE database. (DOCX 15 kb)

Additional file 3: Data extraction form. Sample data extraction for use in the review. (DOCX 15 kb)

## Abbreviations

AJOL: African Journals Online
ASSIA: Applied Social Sciences Index and Abstracts
CERQual: Confidence in the Evidence from Reviews of Qualitative Research
CINAHL: Cumulative Index to Nursing and Allied Health Literature
CRD: Centre for Reviews and Dissemination
ENTREQ: Enhancing transparency in reporting the synthesis of qualitative research
FIGO: International Federation of Gynecology and Obstetrics
GRADE: Grading of Recommendations Assessment, Development and Evaluation
MEDLINE: Medical Literature Analysis and Retrieval System Online
MeSH: Medical subject headings
MMAT: Mixed Methods Appraisal Tool
PICO: Population, Intervention, Control, Outcome
PRISMA: Preferred Reporting Items for Systematic Reviews and Meta-Analyses
PRISMA-P: PRISMA for systematic review protocols
PROSPERO: International prospective register of systematic reviews
USA: United States of America
WHO: World Health Organization
