# Supplementary material for: What influences women’s movement and the use of different positions during labour and birth: a systematic review protocol
Source: Syst Rev. 2018 Nov 13;7:188. doi: 10.1186/s13643-018-0857-8 (PMC6234601; doi:10.1186/s13643-018-0857-8)
Supplement: Supplementary file 2 — MEDLINE search terms. Terms to be used when searching MEDLINE database. (DOCX 15 kb) [file 13643_2018_857_MOESM2_ESM.docx]

**Additional File 2 – Medline search terms**

1. Parturition/
2. Delivery, obstetric/
3. Labor, obstetric/
4. Labour stage, first/
5. Labour stage, second/
6. Labour stage, third/
7. Or/1-6
8. Posture/
9. Patient positioning/
10. Movement/
11. Mov*.ti,ab.
12. Position*.ti,ab.
13. Or/8-12
14. Experience.ti,ab.
15. Report.ti,ab.
16. Account.ti,ab.
17. Story.ti,ab.
18. Stories.ti,ab.
19. Recount*.ti,ab.
20. Affect*.ti,ab.
21. Effect*.ti,ab.
22. Influenc*.ti,ab.
23. Support*.ti,ab.
24. Restrict*.ti,ab.
25. Enabl*.ti,ab.
26. Encourag*.ti,ab.
27. Promot*.ti,ab.
28. Discourag*.ti,ab.
29. Prevent*.ti,ab.
30. Assist*.ti,ab.
31. Persuad*.ti,ab.
32. Dissuad*.ti,ab.
33. Allow*.ti,ab.
34. Able.ti,ab.
35. Unable.ti,ab.
36. Ability.ti,ab.
37. Inability.ti,ab.
38. Inhibit*.ti,ab.
39. Facilitat*.ti,ab.
40. Stop*.ti,ab.
41. Help*.ti,ab.
42. Hinder*.ti,ab.
43. Choice.ti,ab.
44. Choose.ti,ab.
45. Decision.ti,ab.
46. Barrier*.ti, ab.
47. Imped*.ti,ab.
48. Obstacle*.ti,ab.
49. Refus*.ti,ab.
50. Determine*.ti,ab.
51. Restrain*.ti,ab.
52. Or/14-51
53. 7 AND 13 AND 52
54. 53 and “humans” [subjects]

Please note that Medical Subject Heading (MeSH) are indicated here by terms that end in /
