# Supplementary material for: What influences women’s movement and the use of different positions during labour and birth: a systematic review protocol
Source: Syst Rev. 2018 Nov 13;7:188. doi: 10.1186/s13643-018-0857-8 (PMC6234601; doi:10.1186/s13643-018-0857-8)
Supplement: Supplementary file 3 — Data extraction form. Sample data extraction for use in the review. (DOCX 15 kb) [file 13643_2018_857_MOESM3_ESM.docx]

**Additional File 3 – Data extraction form**
